# Overexpression of *Hevea brasiliensis* ethylene response factor *HbERF‐IXc5* enhances growth and tolerance to abiotic stress and affects laticifer differentiation

**DOI:** 10.1111/pbi.12774

**Published:** 2017-09-02

**Authors:** Retno Lestari, Maryannick Rio, Florence Martin, Julie Leclercq, Natthakorn Woraathasin, Sandrine Roques, Florence Dessailly, Anne Clément‐Vidal, Christine Sanier, Denis Fabre, Sémi Melliti, Sony Suharsono, Pascal Montoro

**Affiliations:** ^1^ CIRAD UMR AGAP Montpellier France; ^2^ Universitas Indonesia (UI) Depok Indonesia; ^3^ Bogor Agricultural University (IPB) Bogor Indonesia; ^4^ Faculty of Natural Resources Department of Plant Science Prince of Songkla University (PSU) Hat Yai Songkla Thailand

**Keywords:** genetic modification, latex, plant hormone, rubber, transcription factor

## Abstract

Ethylene response factor 1 (ERF1) is an essential integrator of the jasmonate and ethylene signalling pathways coordinating a large number of genes involved in plant defences. Its orthologue in *Hevea brasiliensis*,* HbERF‐IXc5*, has been assumed to play a major role in laticifer metabolism and tolerance to harvesting stress for better latex production. This study sets out to establish and characterize rubber transgenic lines overexpressing *HbERF‐IXc5*. Overexpression of *HbERF‐IXc5* dramatically enhanced plant growth and enabled plants to maintain some ecophysiological parameters in response to abiotic stress such as water deficit, cold and salt treatments. This study revealed that *HbERF‐IXc5* has rubber‐specific functions compared to *Arabidopsis* ERF1 as transgenic plants overexpressing *HbERF‐IXc5* accumulated more starch and differentiated more latex cells at the histological level. The role of HbERF‐IXc5 in driving the expression of some target genes involved in laticifer differentiation is discussed.

## Introduction

The plant hormone ethylene plays a crucial role in regulating plant growth, development and responses to biotic and abiotic stress (Abeles *et al*., 1992). Ethylene binds to endoplasmic reticulum‐localized receptors that function as negative regulators of ethylene signalling in the absence of the hormone (Chen *et al*., 2002). Ethylene response factors (ERFs) are the last known actors in the ethylene transduction pathway and regulate downstream ethylene‐responsive genes. Members of the ERF transcription factor gene family have at least one conserved AP2 domain of about 60 amino acids. ERFs are trans‐acting factors that bind to GCC, or GCC box, or dehydration‐responsive element (DRE)/C‐repeat motifs (DRE/CRT), *cis*‐acting elements in the promoter region of target genes. Following Nakano's classification, the ERF family was subdivided into ten functional groups (Nakano *et al*., 2006). Group IX is known to have several members involved in ethylene, jasmonate and pathogen responses. Two members of this group, ERF1 and ORA59, are essential integrators of the jasmonate and ethylene signal transduction pathways (Lorenzo *et al*., 2003; Pré *et al*., 2008). Constitutive expression of ERF1 in *Arabidopsis* confers resistance to pathogens and several necrotrophic fungi (Berrocal‐Lobo and Molina, 2004; Berrocal‐Lobo *et al*., 2002; Onate‐Sanchez and Singh, 2002). The *ERF1* gene is used for applications in several cultivated species, notably to improve tolerance to abiotic stress. A patent has been filed by the University of Pennsylvania on this gene and its use in plants (Ecker and Solano, 2002). Constitutive expression of *ERF1* activates the transcription of effector genes encoding basic chitinase (ChiB), thionin 2.1 (Thi2.1) and DEFENSIN 1.2 (PDF1.2) (Brown *et al*., 2003; Manners *et al*., 1998; Solano *et al*., 1998). ERF1 is also a major regulatory factor, independent of ABA and of the proline biosynthesis enzyme P5CS1, which is one of the essential factors for tolerance to water stress (Szabados and Savoure, 2010).

Ethylene and jasmonate play a crucial role in controlling natural rubber production by regulating responses to harvesting stress (Pirrello *et al*., 2014; Putranto *et al*., 2015a,c). *Hevea brasiliensis* is the main commercial source of natural rubber. Natural rubber is produced in laticifers, which constitute an articulated network of latex cells. These cells are differentiated from the cambium and are inserted into the phloem tissue. The latex is harvested by tapping soft bark, and stimulation with ethephon, an ethylene releaser, is used to improve latex flow and latex regeneration between two tappings. Jasmonates and wounding are also factors that induce laticifer differentiation (Hao and Wu, 2000; Tian *et al*., 2015). The ERF family has been identified and classified in *H. brasiliensis* (Duan *et al*., 2013). Two orthologues to *Arabidopsis* ERF1 have been predicted in the rubber tree (Putranto *et al*., 2015a). These genes, *HbERF‐IXc4* and *HbERF‐IXc5*, are highly expressed in bark and latex tissues (Piyatrakul *et al*., 2014). They are also activated in the latex from tapped or ethephon‐stimulated trees, and in leaves of plants grown under abiotic stress (Putranto *et al*., 2015a). A mix of ethylene (in the form of ethylene gas or ethephon), and methyl jasmonate (MeJA) or wounding stress (such as tapping or mechanical wounding known to induce the production of JA) trigger a very strong expression of these genes in *Hevea*, as has been found for the ERF1 orthologue in cotton (Champion *et al*., 2009). Moreover, subcellular localization and transactivation experiments showed that *HbERF‐IXc4* and *HbERF‐IXc5* act as transcriptional activators in the nucleus (Putranto *et al*., 2015a). These results suggest that *HbERF‐IXc4* and *HbERF‐IXc5* play an essential role in latex stress signalling. No major regulation of these genes occurs at the onset of the physiological syndrome known as tapping panel dryness (TPD), which affects latex production (Putranto *et al*., 2015b).

This study sets out to characterize the function of one of the ERF1 orthologue namely *HbERF‐IXc5* gene in a nonmodel species. An efficient *Agrobacterium tumefaciens*‐mediated genetic modification procedure opens up opportunities for further functional analysis in the rubber tree (Blanc *et al*., 2006; Leclercq *et al*., 2010). Although a *Hevea* gene was first inserted in transgenic rubber plants in 2003 (Jayashree *et al*., 2003), the first complete functional analysis of a gene encoding a *Hevea* copper zinc superoxide dismutase was recently carried out in transgenic rubber (Leclercq *et al*., 2012). Based on this effective procedure, the study sets out to produce and characterize transgenic rubber plants overexpressing the *HbERF‐IXc5* gene under the transcriptional control of two promoters, *CaMV 35S* and *Hev2.1* (Montoro *et al*., 2008). Sixteen independent transgenic callus lines were established. Transgenic plants were regenerated and cultured for 1 year after acclimatization. The plants were characterized in terms of growth, anatomy and biochemical compounds. Some ecophysiological parameters were also monitored in response to abiotic stress such as water deficit, cold and salt treatments. These data revealed better tolerance to abiotic stress, suggesting that *HbERF‐IXc5* may be involved in plant defence mechanisms. *HbERF‐IXc5* is assumed to be an integrator of the ethylene and jasmonate signalling pathways. This study also provided new insight into developmental processes, such as latex cell differentiation, which is induced by the plant stress hormone jasmonate.

## Results

### Establishment, plant regeneration and molecular analyses of transgenic callus lines

**Figure 1:**
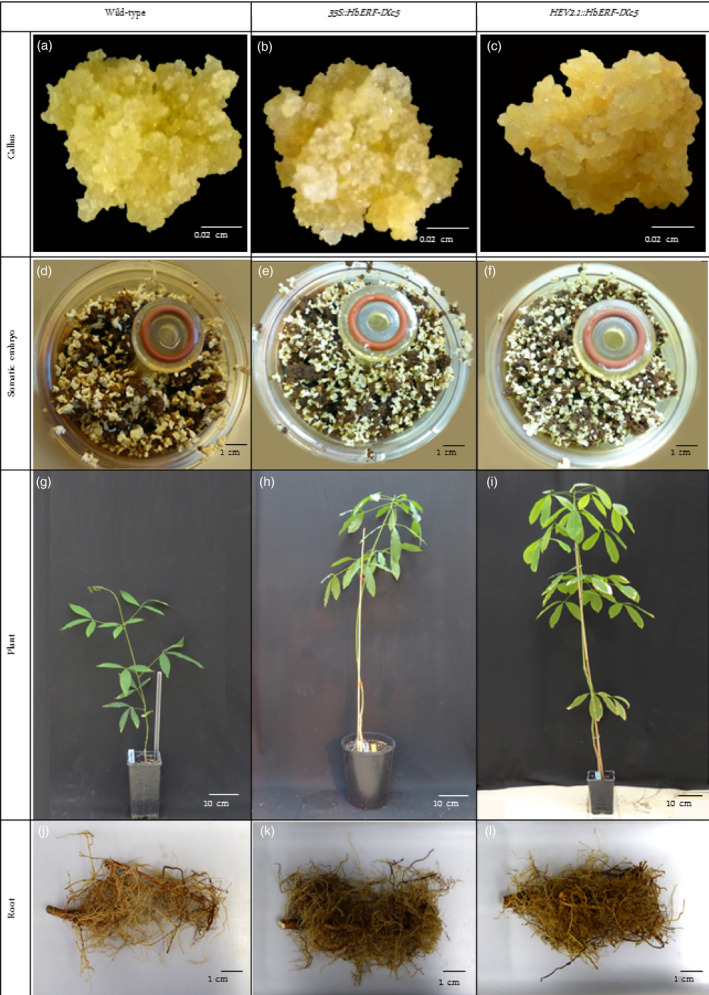
Description of plant regeneration from friable callus lines of wild‐type and *HbERF‐IXc5* transgenic lines. Callus of wild‐type (a), and transgenic lines TS19A90 (b), TS20A75 (c). View of RITA system with brown callus and somatic embryo of wild‐type (d), and transgenic lines TS19A90 (e), TS20A75 (f); 12‐month‐old plant of wild‐type (g), and transgenic lines TS19A90 (h), TS20A75 (i). Root system of wild‐type (j), and transgenic lines TS19A90 (k), TS20A75 (l)

Callus from embryogenic and friable line CI07060 was inoculated with *A. tumefaciens* strain EHA105 harbouring binary vectors *pCamway‐35S::HbERF‐IXc5* and *pCamway‐HEV2.1::HbERF‐IXc5*. After a 5‐day coculture, calli were transferred as small aggregates to the decontamination medium, DM. After a selection phase of 10 subcultures of 3 weeks on DM medium supplemented with paromomycin, 5 and 11 paromomycin‐resistant lines were established from about 300 callus aggregates initially cultured for each construct, respectively (Table 1, Data S1). Promoters directing the *HbERF‐IXc5* gene had an important effect on the number of established lines. The regeneration of somatic embryos and their conversion into plantlets were attempted for each transgenic line and the wild‐type line CI07060. All lines regenerated somatic embryos. The plant regeneration process of stable transgenic lines is described in Figure 1. Of the 16 transgenic lines, 10 were able to produce well‐shaped embryos but only six transgenic lines were able to produce plantlets. The embryo to plantlet conversion rate was very variable between transgenic lines. Lines TS19A90, TS20A69 and TS20A75 showed higher embryogenic capacity than WT. TS20A75 produced the largest number of total somatic embryos (223 per g of callus). On the other hand, TS20A69 achieved the highest production of well‐shaped embryos.

**Table 1:** Plant regeneration ability of wild‐type and *HbERF‐IXc5* transgenic lines. The somatic embryos consisted of two classes: well‐shaped embryos and abnormal embryos. Only well‐shaped embryos were transferred for conversion into plantlets. The conversion percentage was calculated as follows: number of plants/number of well‐shaped somatic embryos

		Replication	Total embryos	Well‐shaped embryo	Plant
Construct	Line	No RITA	No g^−1^ FM	No g^−1^ FM	No g^−1^ FM
None	CI07060	20	58^c^	22^b^	9^abc^
*35S::HbERF‐IXc5*	TS19A46	12	32^c^	7^b^	5^bc^
	TS19A59	5	13^c^	4^b^	1^c^
	TS19A90	14	176^ab^	56^a^	19^ab^
	TS19A99	13	102^bc^	11^b^	0^c^
	TS19A101	7	5^c^	0^b^	0^c^
*HEV2.1::HbERF‐IXc5*	TS20A29	7	12^c^	5^b^	0^c^
	TS20A38	8	4^c^	0^b^	0^c^
	TS20A44	6	17^c^	0^b^	0^c^
	TS20A45	6	10^c^	0^b^	0^c^
	TS20A47	7	12^c^	2^b^	1^c^
	TS20A53	8	24^c^	4^b^	0^c^
	TS20A69	14	176^ab^	82^a^	16^abc^
	TS20A75	15	223^a^	76^a^	24^a^
	TS20A78	5	5^c^	1^b^	0^c^
	TS20A82	10	4^c^	0^b^	0^c^
	TS20A90	6	2^c^	0^b^	0^c^

Statistical analysis was performed with an ANOVA followed by the Tukey test. Values with the same letter were not significantly different at the 0.05 probability level.

**Figure 2:**
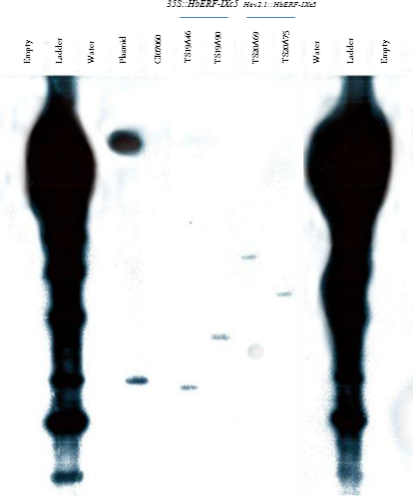
Determination of copy number by Southern blot hybridization. Genomic DNA of leaves were digested with *Eco* RI. The blot was hybridized with a ^32^P radio‐labelled probe corresponding to *NPTII* gene

The four lines producing a sufficient number of plants for further experiments (TS19A46, TS19A90, TS20A69 and TS20A75) had only one copy of T‐DNA according to Southern blot hybridization (Figure 2). A gene expression analysis by real‐time RT‐PCR revealed a significant higher relative transcript abundance in all the transgenic lines compared to WT, which expressed only the native *HbERF‐IXc5* gene (Table 2, Data S2). This confirmed overexpression of the *HbERF‐IXc5* gene in the transgenic lines.

**Table 2:** Relative transcript abundance for the *HbERF‐IXc5* gene in leaves of wild‐type and *HbERF‐IXc5* transgenic lines. The reference gene used was *HbRh2b* (Piyatrakul *et al*. 2014). Each data are the mean of four biological replicates

Construct	Line	Relative transcript abundance
None	WT	1.15 E − 04^c^
*35S::HbERF‐IXc5*	TS19A46	1.98 E − 02^b^
*35S::HbERF‐IXc5*	TS19A90	2.77 E + 00^a^
*Hev2.1::HbERF‐IXc5*	TS20A69	1.23 E + 00^a^
*Hev2.1::HbERF‐IXc5*	TS20A75	3.66 E − 03^b^

Statistical analysis was performed with an ANOVA followed by the Tukey test. Values with the same letter were not significantly different at the 0.05 probability level.

### Monitoring growth and ecophysiological parameters in 1‐year‐old plants

**Figure 3:**
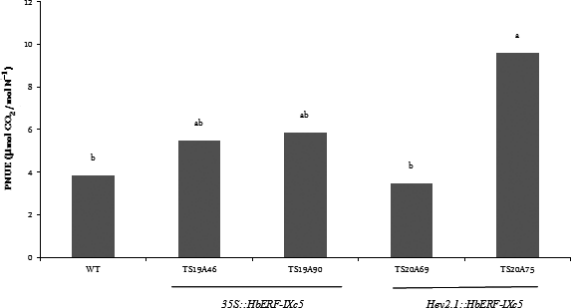
Comparison of photosynthetic nitrogen use efficiency (PNUE, A_400_/Na) between wild‐type and several transgenic lines under optimum growth conditions without water stress. Statistical analysis was performed with an ANOVA followed by the Newman and Keuls test. Values with the same letter were not significantly different at the 0.05 probability level

Several parameters were measured on WT and transgenic plants to quantify plant growth (Table 3, Data S3–S4). All the transgenic lines were significantly more vigorous than the WT line, except line TS19A90. The plants of these lines (TS19A46, TS20A69 and TS20A745) were taller (+15 to 34%) and heavier (+170 to 190%), and had more leaves than the WT plants. Although all parts of the plant were heavier, the root system was comparatively more developed in the transgenic lines (1.6–2.7 times) than in the WT line. A comparison of photosynthetic nitrogen use efficiency (PNUE) revealed that the plants from line TS20A75 maintained significantly greater photosynthetic activity (19.22 μmol CO_2_ mol N^−1^) than the WT plants (5.11 μmol CO_2_ mol N^−1^) under optimum culture conditions (Figure 3, Data S5).

**Table 3:** Growth analysis of 1‐year‐old plants from wild‐type and *HbERF‐IXc5* transgenic lines. Each data item is the mean of 40 replications for morphological and eight replications for weight measurements. Dry matter was measured on eight plants for leaf and stem, and statistical analysis was performed with an ANOVA followed by the Newman–Keuls test

Construct	Line	Number of leaves	Stem diameter (mm)	Stem height (mm)	Dry matter (g/plant)			
					Total	Leaves	Stem	Root
None	CI07060	20.98^c^	6.87^c^	570^c^	32.335^c^	9.114^c^	9.330^b^	13.861^c^
*35S::HbERF‐IXc5*	TS19A46	28.73^b^	9.55^a^	742^a^	87.291^a^	19.794^ab^	30.491^a^	37.006^a^
*35S::HbERF‐IXc5*	TS19A90	19.44^c^	6.48^c^	478^d^	56.584^b^	15.539^b^	19.174^b^	21.871^bc^
*HEV2.1::HbERF‐IXc5*	TS20A69	32.84^a^	9.03^a^	761^a^	87.803^a^	24.619^a^	32.631^a^	30.553^ab^
*HEV2.1::HbERF‐IXc5*	TS20A75	26.68^b^	8.19^b^	657^b^	93.831^a^	23.314^a^	32.834^a^	37.682^a^

Values with the same letter were not significantly different at the 0.05 probability level.

### Quantitative and qualitative analyses of some histological parameters

**Figure 4:**
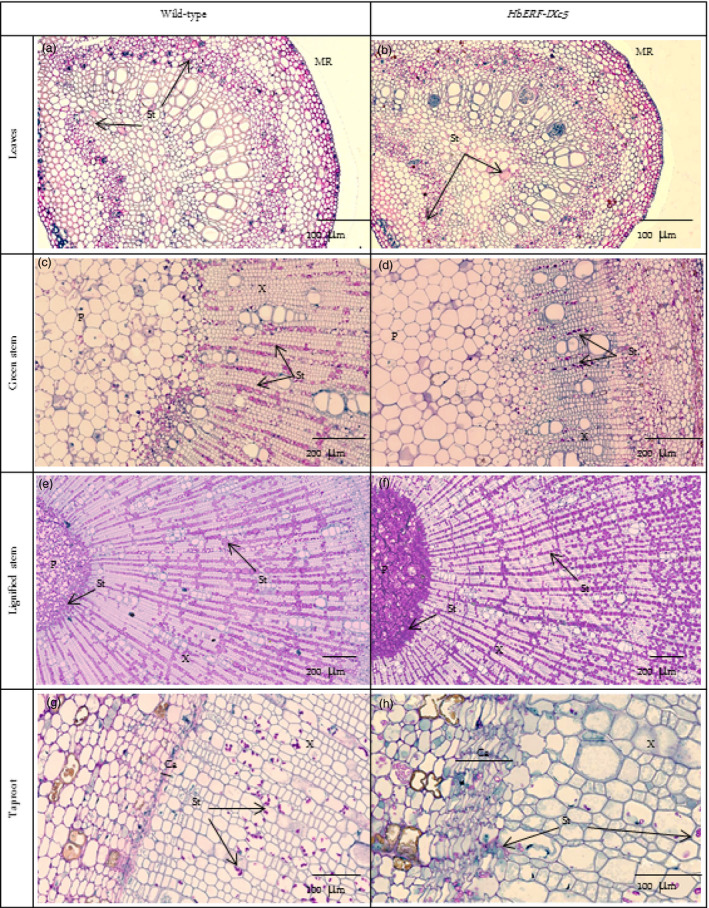
Histological analysis revealing the accumulation of starch reserves. (a) Leaves of wild‐type, (b) Leaves of *HbERF‐IXc5* transgenic lines, (c) Green stem of wild type, (d) Green stem of *HbERF‐IXc5* transgenic lines, (e) Lignified stem of wild type, (f) Lignified stem of *HbERF‐IXc5* transgenic lines, (g) Taproot of wild type, (h) Taproot of *HbERF‐IXc5* transgenic lines. The histological sections were stained with Schiff Naphthol Blue Black. Histological sections were annotated by arrows as follows: MR, midrib; P, pith; X, xylem; Ca, cambium; St, starch

A histological analysis did not reveal any structural changes between WT and transgenic plants (Data S6–S9). No significant differences were noted for leaves (width of cuticle, upper epidermis, palisade parenchyma, lower epidermis and stomata), stem (width of bark, cambium, xylem, pith) and taproot (width of bark, xylem and pith), except for the *Hev2.1::HbERF‐IXc5* lines (TS20A69 and TS20A75) with the wider cambium (73–75 μm) compared to WT (38 μm) in the taproot. However, two striking observations were made. Firstly, a substantial accumulation of starch reserves was especially observed in the lignified part of the stem (Figure 4). Secondly, latex cells were more abundant in the leaf and stem (Table 4).

**Table 4:** Qualitative and quantitative analysis on histological slides of primary and secondary latex cells in leaves, green and lignified stems, and taproot of 1‐year‐old plants from wild‐type and transgenic lines. The abundance of primary latex cells (PCL) and secondary latex cells (SLC) was estimated as follows: (−): absence; (+): rare; (++): 5–10; (+++): 10–50; (++++): >50

Construct	Line	Primary latex cell					Secondary latex cell	
		Leaf		Green stem	Lignified stem	Root	Green stem	Lignified stem
		Midrib	Lamina					
None	CI07060	50^a^	4^a^	+	−	+	+	+++
*35S::HbERF‐IXc5*	TS19A46	157^c^	5^a^	+++	−	+	+++	++++
*35S::HbERF‐IXc5*	TS19A90	37^a^	3^a^	+	−	+	+	++
*HEV2.1::HbERF‐IXc5*	TS20A69	113^b^	12^a^	+	−	+	+	+
*HEV2.1::HbERF‐IXc5*	TS20A75	84^b^	3^a^	++	−	+	+	++

Statistical analysis was performed with an ANOVA followed by the Tukey test. Values with the same letter were not significantly different at the 0.05 probability level.

**Figure 5:**
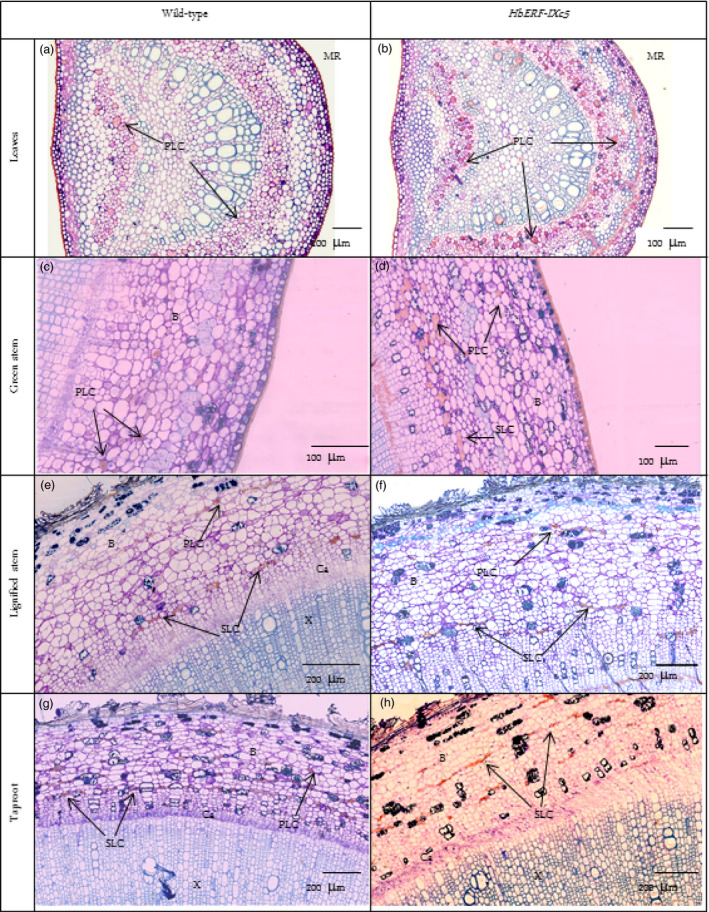
Presence of latex cells in leaves, stem and root of 1‐year‐old plants from wild‐type and *HbERF‐IXc5* transgenic lines. (a) Latex cell in leaves of wild type; (b) Latex cell in leaves of *HbERF‐IXc5* transgenic lines; (c) Latex cell in green stem of wild type; (d) Latex cell in green stem of *HbERF‐IXc5* transgenic lines; (e) Latex cell in lignified stem of wild‐type; (f) Latex cell in lignified stem of *HbERF‐IXc5* transgenic lines; (g) Latex cell in taproot of wild type; (h) Latex cell in taproot of *HbERF‐IXc5* transgenic lines. The histological sections were stained with Oil Red O. Histological sections were annotated by arrows as follows: B, bark; Ca, cambium; PLC, primary latex cells; SLC, secondary latex cell; X, xylem

Primary latex cells could be counted on 2 mm of leaves. Lines TS19A46, TS20A69 and TS20A75 had significantly much more primary latex cells in the midrib of leaves (157, 113 and 84) compared to the WT line (50). A qualitative analysis of primary and secondary latex cells in green and lignified stems revealed a clear abundance of these types of cells in the plants of line TS19A46 (Table 4, Figure 5). Numerous secondary latex cells were randomly distributed, either as isolated cells or in a group not yet forming anastomoses. Laticifer cells had a thick cell wall, nontransparent, elastic cytoplasm and were stained in pink‐red (Figure 5).

### Carbohydrate and antioxidant contents

In an attempt to confirm an accumulation of starch reserves, several carbohydrate compounds were quantified in 1‐year‐old plants growing under standard conditions (Table 5, Data S10). Starch was observed as pink‐red coloured grains stained by periodic acid–Schiff. The starch content increased dramatically in the bark and pith of both green and lignified stems of the transgenic lines compared to WT, but the variability did not lead to significant differences. By contrast, there were no large differences in contents for soluble sugar (glucose, fructose and sucrose). Interestingly, we found that sucrose was the most important carbohydrate in stem bark and in leaves, while the starch content was the highest compound in stem pith. Starch reserves were more abundant in the bark of lignified stems from the transgenic lines compared to the wild type.

**Table 5:** Quantification of starch accumulation in the pith of lignified stem of 1‐year‐old plants from wild‐type and *HbERF‐IXc5* transgenic lines. All values are expressed in mg of each carbohydrate per gram of dry matter except starch content, which was converted into equivalent glucose

Tissue	Construct	Line	Starch	Glucose	Fructose	Sucrose	Soluble sugar
Bark lignified stem	None	CI07060	17^b^	12^b^	6^b^	127^cde^	145^bcd^
	*35S::HbERF‐IXc5*	TS19A90	41^b^	6^b^	3^b^	131^cde^	140^bcd^
	*HEV2.1::HbERF‐IXc5*	TS20A69	79^b^	8^b^	4^b^	140^cde^	152^bcd^
	*HEV2.1::HbERF‐IXc5*	TS20A75	99^b^	7^b^	4^b^	128^cde^	139^bcd^
Pith lignified stem	None	CI07060	147^ab^	2^b^	1^b^	36^de^	39^e^
	*35S::HbERF‐IXc5*	TS19A90	68^b^	1^b^	0^b^	33^e^	34^e^
	*HEV2.1::HbERF‐IXc5*	TS20A69	266^a^	1^b^	0^b^	47^de^	48^de^
	*HEV2.1::HbERF‐IXc5*	TS20A75	125^b^	1^b^	1^b^	32^e^	34^e^
Bark green stem	None	CI07060	8^b^	31^b^	3^b^	148^bcde^	182^bc^
	*35S::HbERF‐IXc5*	TS19A90	21^b^	23^b^	2^b^	128^cde^	153^bcd^
	*HEV2.1::HbERF‐IXc5*	TS20A69	35^b^	19^b^	7^b^	152^bcde^	178^bc^
	*HEV2.1::HbERF‐IXc5*	TS20A75	73^b^	22^b^	3^b^	159^bcd^	184^b^
Pith green stem	None	CI07060	23^b^	11^b^	11^b^	48^de^	70
	*35S::HbERF‐IXc5*	TS19A90	74^b^	13^b^	0^b^	80^de^	93^cde^
	*HEV2.1::HbERF‐IXc5*	TS20A69	159^ab^	8^b^	8^b^	51^de^	67^de^
	*HEV2.1::HbERF‐IXc5*	TS20A75	108^b^	10^b^	11^b^	61^de^	82^de^
Leaves	None	CI07060	7^b^	82^a^	18^ab^	249^ab^	349^a^
	*35S::HbERF‐IXc5*	TS19A90	9^b^	78^a^	18^ab^	285^a^	381^a^
	*HEV2.1::HbERF‐IXc5*	TS20A69	3^b^	62^ab^	24^ab^	227^abc^	313^a^
	*HEV2.1::HbERF‐IXc5*	TS20A75	19^b^	99^a^	47^a^	211^abc^	357^a^

Analysis was performed with an ANOVA followed by the Newman–Keuls test. Values with the same letter were not significantly different at the 0.05 probability level.

**Figure 6:**
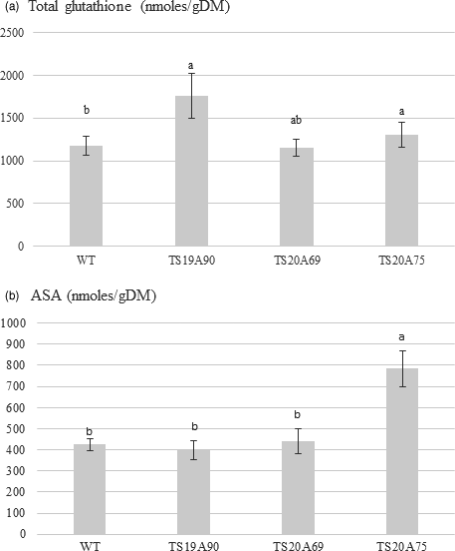
Antioxidant contents in leaves of 1‐year‐old plants from wild‐type (WT) and transgenic lines. (a) Total glutathione. (b) Ascorbic acid content (ASA)

*HbERF‐IXc5* is assumed to control the expression of genes involved in ROS‐scavenging systems. For that reason, two main antioxidant compounds were quantified in leaves of 1‐year‐old plants growing under standard conditions (Figure 6, Data S11). Total glutathione contents ranged from 1150 to 1760 nmoles/gDM. Two transgenic lines TS19A90 and TS20A75 had a significant greater content than the control (Figure 6a). The ascorbic acid content was about 400 nmoles/gDM in WT and in lines TS19A90 and TS20A69, and peaked at 783 nmoles/gDM for line TS20A75 (Figure 6b).

### Effect of abiotic stress

**Figure 7:**
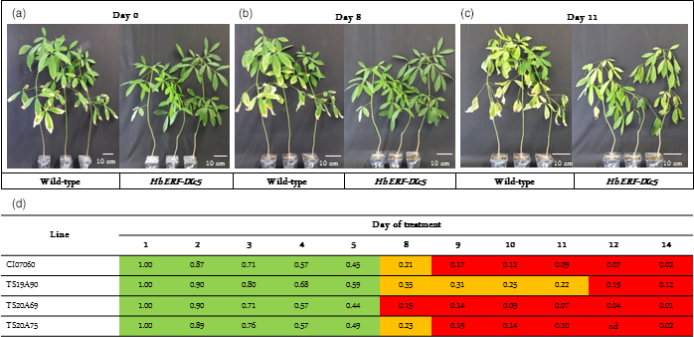
Effect of water deficit on 8‐month‐old plants from wild‐type and transgenic lines and their FTSW. (a) Plants before water stress on day 0 (FTSW about 1). (b) Plants after 8 days of water deficit (FTSW ≥ 0.2). (c) Plants after 14 days of water deficit (FTSW < 0.2). (d) Daily change in FTSW value after water deficit treatment. Highlighting in green (1 > FTSW > 0.4), in orange (0.4 > FTSW > 0.2), in red (FTSW < 0.2). nd: nondetermined

In order to test tolerance to abiotic stress, plants were subjected to water deficit, salt and cold treatments. Up to 8 days after water deficit, plants had turgescent leaves (Figure 7a,b). Fourteen days after no watering, both WT and transgenic plants displayed the same leaf wilting symptoms (Figure 7c). In the absence of an obvious phenotype, the water deficit effect was evaluated by monitoring the fraction of transpirable soil water (FTSW) (Data S12, Figure 7d). Moderate and severe physiological stress statuses were considered for FTSW below 0.4 and 0.2, respectively. Moderate and severe stress status was reached for plants of all lines within 8 and 9 days after no watering, respectively, revealing that moderate stress status was very transient except for line TS19A90, which maintained this status for 4 days.

**Figure 8:**
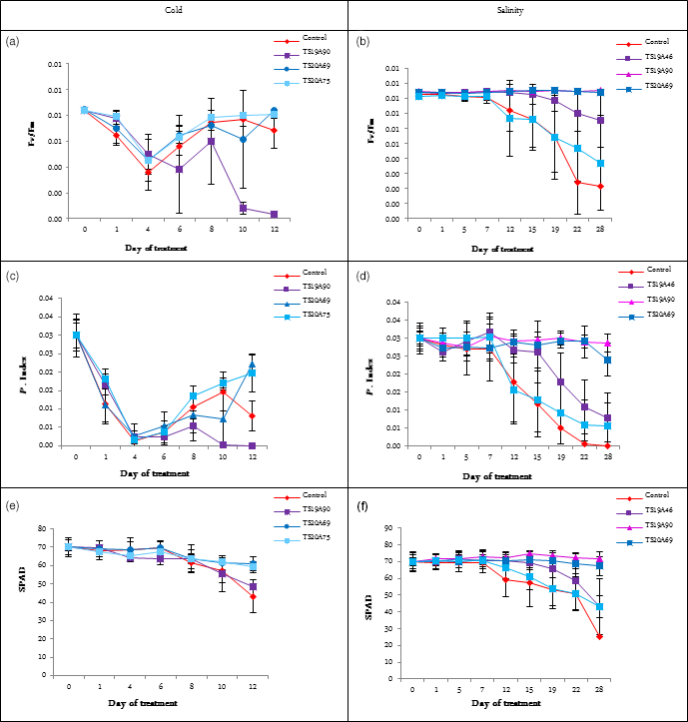
Effect of cold and salinity stress on various ecophysiological parameters for wild‐type and several transgenic lines. (a) Effect of cold stress on Fv/Fm value. (b) Effect of salinity stress on Fv/Fm value. (c) Effect of cold stress on P. Index value. (d) Effect of salinity stress on P. Index value. (e) Effect of cold stress on SPAD value. (f) Effect of salinity stress on SPAD value. Statistical analysis was performed with an ANOVA followed by the Tukey test. Values with the same letter were not significantly different at the 0.05 probability level

The Fv/Fm and PI values decreased dramatically during the 4 days of cold treatment and then increased for all lines except TS1A90 (Figure 8a,c). Overall, TS20A75 showed the highest average Fv/Fm values, while TS20A69 and TS20A75 showed the highest average PI values. Higher Fv/Fm and PI values indicated the high tolerance to cold stress (Kadir *et al*., 2007). By contrast, the chlorophyll content (SPAD) decreased slightly during and after treatment (Figure 8e). All lines showed a higher SPAD value compared to the wild type.

As regards the salinity treatment, the Fv/Fm and PI values showed that WT plants, presenting the lowest values, were more affected than transgenic lines after 28 days of treatments (Figure 8b,d). This indicates that the photosystem II capacity was degraded in WT plants. At the same time, the chlorophyll content decreased particularly in WT plants (Figure 8f). TS19A90 and TS20A69 were found to perform better and had a more stable chlorophyll content. Both lines also gave the highest SPAD value during the treatment. This suggests that transgenic plants may absorb light and accumulate energy better and thus avoid oxidative damage. The PI values peaked for lines TS19A90 and TS20A69. This result suggests that these transgenic lines might delay leaf senescence under high salinity concentrations, which is considered to be an important and favourable trait, known as ‘stay‐green’.

## Discussion

*HbERF‐IXc5* belongs to group IX of the ERF transcription factor family (Duan *et al*., 2013). This gene shares several common functional features of its putative orthologous gene *ERF1* (Duan *et al*., 2013; Putranto *et al*., 2015a). Interestingly, this study revealed several new characteristics of rubber transgenic lines overexpressing *HbERF‐IXc5* in terms of somatic embryogenesis, growth, starch reserves and laticifer differentiation, as well as better tolerance to abiotic stress.

### Overexpression of *HbERF‐IXc5* affects the somatic embryogenesis process

Overexpression of the *HbERF‐IXc5* gene induced greater variability in callus proliferation, production of somatic embryos and plant regeneration capacity (Table 1, Figure 1). Callus browning was correlated with somatic embryogenesis induction (Lardet *et al*., 2009), but the *HbERF‐IXc5* callus lines turned very rapidly brown, almost necrotic, in response to the low growth regulator content in the EXP medium. This feature had a negative impact on the production of somatic embryos for several lines. By contrast, a small number of lines could produce a larger number of embryos and plantlets compared to the wild‐type line. This observation led to the conclusion that there is no deleterious effect of this gene whatever the stages of development. Use of the *HEV2.1* promoter to direct the *HbERF‐IXc5* gene reduced the negative effect of the transgene with a larger number of established transgenic lines. ROS‐scavenging system modified transgenic lines remained yellow throughout the somatic embryogenesis process, and they mostly lost their ability to regenerate somatic embryos and plantlets (Leclercq *et al*., 2012). Indeed, ROS detoxification enzymes and ethylene play an important role in somatic embryogenesis of woody species (Gomez‐Garay *et al*., 2013; Jo *et al*., 2014). This suggests that *HbERF‐IXc5* might control several target genes triggering a metabolic response related to the oxidation of polyphenols and did not produce a sufficient amount of antioxidants to protect cells.

Stress in plant tissue cultures induces ethylene production, and consequent negative effects in terms of tissue browning. But it can also promote growth and cell proliferation depending on the type of tissue, stage of development and environmental factors (Kepczynska and Zielinska, 2011; Pierik *et al*., 2006). ERFs coordinate ethylene response. Among the differentially expressed *ERF* genes at different stages of the *Hevea* somatic embryogenesis process (Piyatrakul *et al*., 2012), the *HbERF‐IXc4* gene was highly expressed in callus during embryogenesis induction in highly embryogenic lines and down‐regulated in nonembryogenic lines. By contrast, its paralogue *HbERF‐IXc5* was not regulated at all during the somatic embryogenesis process. These data suggest that an overexpression of the *HbERF‐IXc5* gene might induce, among others, *HbERF‐IXc4* target genes involved in somatic embryogenesis. Nevertheless, variability in the response of *Hevea* tissue could indicate that this factor is not the main one and may well cooperate with others in fine‐tuning this biological process.

### Overexpression of *HbERF‐IXc5* improves the vigour of plant material and influences photosynthetic activity

Transgenic plants from the *HbERF‐IXc5* lines had better growth than the wild‐type plants except line TS19A90 (Table 3). The better performance of the *HbERF‐IXc5* transgenic lines can be attributed to several features. Firstly, the root system was well developed and could contribute to better nutrient and water uptake. Secondly, these transgenic plants had more leaves and this well‐developed canopy may have contributed to a strong photosynthetic capacity. This is especially the case for line TS20A75 harbouring *HEV2.1::HbERF‐IXc5*, which had a twofold intrinsic photosynthetic nitrogen use efficiency (PNUE) compared to the WT plants (Figure 3). Thirdly, all the transgenic lines tended to accumulate more starch reserves and, for line TS20A75, more ascorbic acid and total glutathione. The presence of starch could be an indirect indicator of photosynthetic activity (Geigenberger *et al*., 2011). Rubber trees adjust the amount of carbohydrate reserves stored to the level of metabolic demand for latex regeneration at the expense of growth (Silpi *et al*., 2007).

The performance of the *HbERF‐IXc5* lines was quite singular. ERF1‐overexpressing plants showed an extreme dwarf phenotype similar to that of the constitutive ethylene response mutant *ctr1* and *EIN3/EIL1*‐overexpressing transgenic plants. However, this effect on growth can differ in some other reports. Interaction between the environment and the ethylene signalling pathway may affect growth regulation (Vahala *et al*., 2013). Moreover, root growth inhibition by ethylene was reported in overexpressing *ERF1* lines (Mao *et al*., 2016). This analysis also suggested that *HbERF‐IXc5* may have an effect other than cell elongation and development when compared to *ERF1*. Plants from line TS19A90 cannot be considered as dwarf plants but simply smaller plants than control. The expression of *HbERF‐IXc5* in leaves was much greater in transgenic lines than in the control line and peaked for line TS19A90. Position effect of transgene into the genome and promoters may have a significant impact on transgene expression. Expression of *HbERF‐IXc5* gene is under the control of CaMV 35S promoter in line TS19A90. Hevein2.1 is a major latex protein having laticifer‐specific expression in roots and stems, so the promoter of this gene can be considered as a strong promoter. Finally, this promoter is also induced by light and consequently can drive transgene expression in leaves (Montoro *et al*., 2008). For that reason, these two promoters can drive strong gene expression in leaves. Transgene dose effect could be observed in nonphotosynthetic tissues such as callus (impact on somatic embryogenesis) and bark (impact on laticifer differentiation).

### Overexpression of *HbERF‐IXc5* improves tolerance to abiotic stress

The *HbERF‐IXc5* lines were screened for their tolerance to water deficit, cold and salinity treatments. *HbERF‐IXc5*‐overexpressing plants did not show any obvious morphological characteristics of tolerance to abiotic stress within the duration of the treatments, but several ecophysiological parameters revealed better stability of leaf physiological status. Similarly to *Arabidopsis* ERF1, HbERF‐IXc5 may be involved in controlling responses to abiotic stress. ERF1 up‐regulates specific target genes in response to biotic and abiotic stress by stress‐specific binding to GCC or DRE/CRT *cis*‐acting elements (Cheng *et al*., 2013). ERF1 positively regulates *Arabidopsis* resistance to the pathogens *Botrytis cinerea* and *Plectosphaerella cucumerina* by binding GCC boxes in defence‐related genes (Berrocal‐Lobo *et al*., 2002; Solano *et al*., 1998). Conversely, ERF1 binds to DRE motifs but not GCC boxes in response to abiotic stress. Transactivation experiments revealed that *HbERF‐IXc5* can activate a GCC box, but no information is available on its action on DRE (Putranto *et al*., 2015a).

Upstream, expression of the *HbERF‐IXc5* gene was induced by dehydration and down‐regulated by cold in rubber juvenile plants (Putranto *et al*., 2015a). In *Hevea* transgenic lines, overexpression of the *HbERF‐IXc5* transgene ultimately gave the same effect for both the salinity and cold treatments, even though the native gene was down‐regulated by the cold treatment. Plant defence mechanisms usually include a strengthening of ROS‐scavenging systems. This study showed an increase in antioxidant contents in some *HbERF‐IXc5*‐overexpressing lines. Line TS20A75 accumulated in plant leaves both more total glutathione and ascorbic acid than other lines. By contrast, a strong browning of transgenic callus lines was noted after somatic embryogenesis induction. This suggests that *HbERF‐IXc5* gene induction by stress may not lead to a sufficient activation of genes involved in ROS‐scavenging systems.

*HbERF‐IXc4* and *HbERF‐IXc5* are assumed to be involved in the crosstalk of the ethylene and jasmonate signalling pathways for several reasons. Firstly, *HbERF‐IXc4* and *HbERF‐IXc5* belong to ERF group IX and it is orthologous to *Arabidopsis* ERF1, which is an integrator of JA and ET signals in plant defence (Lorenzo *et al*., 2003). Secondly, *HbERF‐IXc4* and *HbERF‐IXc5* are controlled by multiple harvesting stresses, tapping and ethephon stimulation, related to jasmonate and ethylene production, respectively (Putranto *et al*., 2015a). Lastly, expression of the *HbERF‐IXc4* and *HbERF‐IXc5* genes was induced by wounding, methyl jasmonate and ethylene, but it was dramatically enhanced by a combination of MeJA and ET like ERF1 (Putranto *et al*., 2015a). The involvement of JA and ET signalling was reported in salt stress induction (Cheng *et al*., 2013). Taken together, these data suggest that *HbERF‐IXc5* contributes to the implementation of defence mechanisms in the rubber tree but not necessarily through the sensing of cell redox homoeostasis and the control of antioxidant production. Further characterization of transgenic *HbERF‐IXc4* lines will allow to confirm its involvement in stress tolerance as *HbERF‐IXc5*.

### Overexpression of *HbERF‐IXc5* induces laticifer differentiation

*HbERF‐IXc5*‐overexpressing plants had significantly more primary latex cells in the midrib of leaves compared to WT. For line TS19A46, both primary and secondary latex cells were more abundant in green and lignified stems (Table 4). This suggests that *HbERF‐IXc5* may contribute to the laticifer differentiation mechanism. Primary latex cells are isolated within parenchymatous tissues when secondary laticifers are differentiated from vascular cambium. Activation of cambium was found in the *Hevea* transgenic lines. ERFs are essential for ethylene‐stimulated cambial cell division (Etchells *et al*., 2012). Overexpression of some ERFs can stimulate diameter growth in *Populus* plants (Vahala *et al*., 2013). Young stems had primary latex cells and no secondary laticifers, with the latter appearing after development of five extension units (Hao and Wu, 1982). However, a large number of secondary laticifers were observed in young green stems of line TS19A46 revealing an earlier occurrence than in wild‐type plants. Tapping and mechanical wounding were first shown to activate laticifer differentiation (Hao and Wu, 1982). Then, jasmonates were identified as factors involved in laticifer differentiation induced by mechanical wounding (Hao and Wu, 2000; Tian *et al*., 2003; Wu *et al*., 2002). When applied to extending young green stems, jasmonates led to a significant increase in the number of primary laticifers but did not induce secondary laticifer differentiation (Hao and Wu, 2000). Secondary laticifer differentiation was obtained by jasmonate application to the extended young stems. Ethephon caused no obvious change in the number of laticifer rings when applied to trees without latex exploitation (Hao and Wu, 1984). This suggests that ethylene does not play a direct role in laticifer differentiation. By contrast, interactions between jasmonic acid (JA) and cytokinin or the stress signal pathway mediate wound‐induced secondary laticifer differentiation in rubber trees (Tian *et al*., 2015; Zhang and Tian, 2015; Zhang *et al*., 2015). Intensive studies on the identification of differentially expressed genes and signalling pathways should lead to a better understanding of secondary laticifer differentiation (Hong *et al*., 2015; Loh *et al*., 2016).

Several *AP2/ERF* genes (*HbERF1, HbERF2, HbERF3* and *RAV1*) were induced by JA in bark during JA‐induced laticifer differentiation (Wu *et al*., 2010). *HbERF1*,* HbERF2* and *HbERF3* corresponded to *HbERF‐VIIa3*,* HbERF‐VIIa17* and *HbERF‐VIIa1* according to the *Hevea AP2/ERF* superfamily classification (Duan *et al*., 2013). The ERFs from group VII are known to regulate hypoxia‐responsive genes (Licausi *et al*., 2013). Although *HbERF‐IXc5* was not identified in JA‐induced laticifer differentiation, *HbERF‐IXc5* might be at the crosstalk of the ET and JA signalling pathways and control some target genes directly involved in this biological process.

## Conclusions

This study is the first successful functional analysis of a transcription factor in transgenic lines from *Hevea brasiliensis,* a nonmodel perennial latex‐producing species. *HbERF‐IXc5*‐overexpressing lines turned rapidly brown after somatic embryogenesis induction. Of the sixteen independent transgenic callus lines, sufficient numbers of plants were regenerated from four lines for further characterization. Overexpression of *HbERF‐IXc5* enhanced growth and tolerance to abiotic stress in rubber plants. *HbERF‐IXc5* might control some target genes involved in carbohydrate metabolism, which is an essential feature for latex production. This study also provided new insight into environmentally controlled developmental processes such as laticifer differentiation. Through its role as an integrator of the ethylene and jasmonate signalling pathways, *HbERF‐IXc5* might play a role in JA‐induced laticifer differentiation. Given that *HbERF‐IXc5* should have pleiotropic effects, further characterization will be needed to identify its target genes associated with this biological process. Characterization in transgenic lines of its paralogue *HbERF‐IXc4* should also improve our understanding of the specific role of ERF1 orthologues in *Hevea*.

## Experimental procedures

### Establishment of transgenic callus lines and plant regeneration

The *HbERF‐IXc5* gene was cloned in two *pCamway* binary destination vectors harbouring the *35S CaMV* and latex‐specific *HEV2.1* promoters, respectively (Leclercq *et al*., 2015). These binary vectors were introduced into *A. tumefaciens* strain EHA105 by electroporation. For inoculation, bacteria were grown in liquid Lysogeny Broth medium (Duchefa, Haarlem, The Netherlands) supplemented with 50 mg L^−1^ kanamycin and 100 μm acetosyringone at 28 °C until OD_600 nm_ = 0.6 (Blanc *et al*., 2006). Transgenic lines were established according to the procedure described by Blanc and Leclercq (Blanc *et al*., 2006; Leclercq *et al*., 2010). The production of embryos and their conversion into plantlets were carried out as described by Lardet and coll. (Lardet *et al*., 1999, 2007). Plantlets were then acclimatized in the greenhouse at 28 °C with 60% relative humidity.

### Acclimatization and plant morphology measurements

Plantlets were transferred to 120‐mL paper pots (Jiffy pot, Ohio) and covered with a plastic box. After 2 months of acclimatization, plants were transferred to 2‐L plastic pots for 1 year of growth. Several parameters were measured on plants before acclimatization (length of taproot, diameter of taproot, height of stem, diameter of stem, number of leaves, number of leaflets and number of lateral roots, 2 and 6 months after acclimatization (height of plant, diameter of stem at the collar, number of leaves, and number of leaflets) and then 12 months after acclimatization (diameter of stem, height of stem, number of leaves and leaflets, fresh and dry weight of leaves, stem, total root and main root).

### Molecular analyses

Isolation of genomic DNA and Southern blot hybridization was carried out as described by Leclercq and coll. (Leclercq *et al*., 2010). Hybridization was performed using random primed ^32^P radio‐labelled probes corresponding to *NPTII* genes amplified with the following primers: NPTII‐F: 5′‐CCGGCTACCTGCCCATTCGA‐3′ and NPTII‐R: 5′‐GCGATAGAAGGCGATGCG‐3′. The numbers of bands reflected the number of T‐DNA insertions.

RNA extraction using the caesium chloride cushion and gene expression analysis by real‐time RT‐PCR were carried out according to the methods described by Duan and coll. (Duan *et al*., 2010). This experiment followed recommendations specified by (Udvardi *et al*., 2008). *HbRH2b* was selected from eleven housekeeping genes as the best reference gene according to its stability in tissues from various treatments in juvenile plants and mature trees (Putranto *et al*., 2015b). Real‐time RT‐PCR analysis was carried out using a Light Cycler 480 (Roche, Basel, Switzerland). The transcript abundance level for each gene was relatively quantified by normalization with the transcript abundance of the reference *HbRH2b* gene. Gene *HbERF‐IXc5* was amplified with primers *HbERFCL13262‐F381* 5′‐CAGTTGAAAGAGTGAAGGAATC‐3′ and *HbERFCL13262‐R567* 5′‐TCCAAGTAATCAGCACCCAAG‐3′. Relative transcript abundance took into account primer efficiencies. All the normalized ratios corresponding to transcript accumulation were calculated automatically by Light Cycler Software version 1.5.0 provided by the manufacturer using the following calculation: Normalized Ratio = Efficiency^−Δ(Cp target‐Cp RH2b)^.

### Histo‐cytological analysis

Leaf, green stem, lignified stem and taproot samples followed the same process described in Montoro and coll. (Montoro *et al*., 2008). Sections were stained with two staining treatments: Oil Red O‐Toluidine Blue staining to identify lipid compounds such as latex in red‐orange, lignins, sclerenchyma and polyphenols in blue‐green (Lillie and Ashburn, 1943), and periodic acid–Schiff/Naphthol Blue Black (NBB) to specifically stain walls and polysaccharide storage (starch) in violet, and proteins in blue (Fisher, 1968). To validate the detection of latex cells, stem samples were placed in an ethanol/acetic acid fixative buffer then treated with denaturant agent (iodine bromide) according to the Shi and Hu's method (Shi and Hu, 1965). Samples were directly cut by a microtome into thick section of 40 μm. Rubber inclusions in latex cells appeared in brown and red colours, respectively, for iodine bromide and oil red O stainings (Figure S1).

### Quantification of carbohydrates

Leaves and both the bark and pith of green and lignified stems were collected from wild‐type and transgenic plants and placed quickly in liquid nitrogen. Samples were freeze‐dried and powdered with a laboratory mill to obtain a particle size >100 μm. Sugars were determined by the HPAEC‐PAD technique (ThermoFisher Scientific, Waltham, Massachusetts, USA) with a CarboPack PA1 column and isocratic conditions (150 mm sodium hydroxide) (Mialet‐Serra *et al*., 2005). Starch was quantified by enzymatic assay using a spectrophotometer (Mialet‐Serra *et al*., 2005). Glucose was quantified as described by Boehringer using hexokinase and glucose 6 phosphate dehydrogenase, followed by spectrophotometry of NADPH at 340 nm (Boehringer, 1984).

### Colorimetric measurement of antioxidants

Half a gram of leaves was ground in liquid nitrogen and mixed in 5 mL of 3% sulphosalicylic acid at 4 °C. Samples were centrifuged twice at 11 000 ***g*** for 15 min at 4 °C. Determination of ascorbic acid (ASA) and total glutathione content in leaves was performed on the supernatant according to the method published by Masato and Queval, respectively (Masato, 1980; Queval and Noctor, 2007).

### Application of environmental stress

For the water deficit treatment, 1‐year‐old plants were cut back and placed in pots with 250 g of mould substrate according to the method described by Sanier and coll. (Sanier *et al*., 2013b). Once the plants had developed two growth units, the pots were sealed to prevent soil evaporation and were not watered anymore. The fraction of transpirable soil water (FTSW) was measured and calculated as described by Luquet and coll. (Luquet *et al*., 2008). The FTSW threshold indicates the timing of stomatal closure in response to a soil water deficit (Ray *et al*., 2002; Sinclair and Ludlow, 1986). The experiment ended when the transpiration rate of each stress pot was less than 10% of that of the fully watered pots (Sinclair and Ludlow, 1986). FTSW was decreased from 1 (normal water status) to 0.4 (moderate stress) and 0.2 (severe stress) (Sanier *et al*., 2013a).

For the cold treatment, 1‐year‐old plants growing in 2‐L pots were transferred from the greenhouse at 28 °C to a climatic room at 10 °C for 96 h with a photoperiod of 8 h/16 h. After cold treatment, plants were placed in a room at 20 °C for 1 day, and then transferred to a greenhouse at 28 °C.

For the salinity treatment, plants were watered with 1 litre of a 500 mm sodium chloride solution. Each pot was watered once a week to prevent any increase in osmotic potential from salt.

### Eco‐physiological measurements

For wild‐type and transgenic plants grown under standard conditions, leaf photosynthesis was measured on a mature leaf (stage D) with a portable photosynthesis system (GFS‐3000, Walz, Germany). The measurements were made *in situ* between 9:00 am and 11:00 am at a light intensity of 1200 μmol m^2^.s^−1^ of PAR, with a CO_2_ concentration maintained at 400 ppm, a controlled leaf temperature at 28 °C, a relative humidity in the cuvette set at 65% and a constant flow rate through the cuvette of 800 mL min^−1^. The measured variables were calculated according to von Caemmerer & Farquhar's method (von Caemmerer and Farquhar, 1981), and included leaf net CO_2_ assimilation *(A)*,* Gs* (stomatal conductance), the transpiration rate (*E*) and the intercellular CO_2_ mole fraction (*Ci*). Photosynthetic nitrogen use efficiency was computed (PNUE), expressed as the ratio between net CO_2_ assimilation and leaf nitrogen content (A_400_/Na). The leaves used for gas exchange measurements were also used to determine nitrogen content in the leaves as a percentage of the dry weight (DW; Nm in mg g^−1^ DW), SLA, (cm^2^ g^−1^), leaf mass per area (LMA, the inverse of SLA in g.cm^2^) and, accordingly, nitrogen concentration on a leaf area basis multiplying Nm by LMA (Na in gN m^2^). Total leaf nitrogen was analysed based on the Dumas combustion method using a LECO FP‐528 nitrogen analyser, in the CIRAD plant analysis laboratory. Chlorophyll content was also measured on the same leaf using a SPAD‐502 (Minolta, Ltd., Tokyo, Japan).

For cold and salinity stress, chlorophyll fluorescence measurements were performed using a Handy‐PEA fluorometer (Hansatech Instrument, King's Lynn, Norfolk,VA) on control and stressed plants in the morning on mature leaves up to 12 days after treatment. The fluorescence transients were induced by 1‐s illumination with an array of six light‐emitting diodes providing a maximum light intensity of 3000 μmol (photons) m^−2^ s^−1^ and uniform irradiation over a 4‐mm‐diameter leaf area. Fast fluorescence kinetics (F0 to FM) were recorded from 10 μs to 1 s. The fluorescence intensity at 50 μs was considered as F0 (Strasserf *et al*., 1995). Readings were taken on the abaxial side of mature leaves, dark adapted with a lightweight plastic leaf clip for 30 min before measurement. Maximum photosystem II (PSII) photochemical efficiency *F*_v_ */F*_m_, the ratio of variable fluorescence (*F*_v_) to maximum fluorescence (*F*_m_) and the performance index (*PI*) plant vitality indicator (Strasserf *et al*., 1995; Strauss *et al*., 2006) were calculated automatically. As for photosynthesis measurement, the chlorophyll content value was measured on the same leaf using a SPAD‐502 (Minolta, Ltd., Tokyo Japan).

### Database and statistical analysis

All the experiments comprised several biological replications which are specified in the tables of the results section. The data were normalized prior to statistical analysis using XLSTAT (Addinsoft, Paris, France). Raw data were analysed by ANOVA with various statistical tests. The significance of values was determined for a *P*‐value under 0.05.

## Conflict of interest

Co‐authors declare they have no conflict of interest.

## Supporting information


**Figure S1** Comparison of latex cell‐specific staining using (a) iodine bromide treatment (Shi and Hu, 1965) and (b) oil red O (Montoro *et al*., 2008). Latex cells (LC) are specified by arrows and appear in brown and red for the two stainings, respectively.

**Data S1** Number of GFP positive aggregates during the selection of transgenic lines.
**Data S2** HbERF‐IXc5 gene expression.
**Data S3** Growth raw data for each acclimatized plants.
**Data S4** Plant weight raw data for each acclimatized plants.
**Data S5** Ecophysiological raw data for each analysed plants.
**Data S6** Quantitative and qualitative histological raw data for leaves of each analysed plants.
**Data S7** Quantitative and qualitative histological raw data for green stem of each analysed plants.
**Data S8** Quantitative and qualitative histological raw data for lignified stem of each analysed plant.
**Data S9** Quantitative and qualitative histological raw data for taproot of each analysed plant.
**Data S10** Carbohydrate content raw data for each analysed plants.
**Data S11** Antioxidant content raw data for each analysed plants.
**Data S12** Fraction of transpirable soil water (FTSW) raw data for each analysed plants.

## References

[pbi12774-bib-0001] Abeles, F.B. , Morgan, P.W. and Saltveit, M.E. (1992) Ethylene in Plant Biology. San Diego, California: Academic Press Inc.

[pbi12774-bib-0002] Berrocal‐Lobo, M. and Molina, A. (2004) Ethylene response factor 1 mediates Arabidopsis resistance to the soilborne fungus Fusarium oxysporum. Mol. Plant Microbe Interact. 17, 763–770. 15242170 10.1094/MPMI.2004.17.7.763

[pbi12774-bib-0003] Berrocal‐Lobo, M. , Molina, A. and Solano, R. (2002) Constitutive expression of ETHYLENE‐RESPONSE‐FACTOR1 in Arabidopsis confers resistance to several necrotrophic fungi. Plant J. 29, 23–32. 12060224 10.1046/j.1365-313x.2002.01191.x

[pbi12774-bib-0004] Blanc, G. , Baptiste, C. , Oliver, G. , Martin, F. and Montoro, P. (2006) Efficient *Agrobacterium tumefaciens*‐mediated transformation of embryogenic calli and regeneration of *Hevea brasiliensis* Mull Arg. plants. Plant Cell Rep. 24, 724–733. 16136315 10.1007/s00299-005-0023-3

[pbi12774-bib-0005] Boehringer, S.A. (1984) Methods of Enzymatic Food Analysis using Single Reagents. Germany: Mannheim.

[pbi12774-bib-0006] Brown, R.L. , Kazan, K. , McGrath, K.C. , Maclean, D.J. and Manners, J.M. (2003) A role for the GCC‐box in jasmonate‐mediated activation of the PDF1.2 gene of Arabidopsis. Plant Physiol. 132, 1020–1032. 12805630 10.1104/pp.102.017814 PMC167040

[pbi12774-bib-0007] von Caemmerer, S. and Farquhar, G.D. (1981) Some relationships between the biochemistry of photosynthesis and the gas exchange of leaves. Planta, 153, 376–387. 24276943 10.1007/BF00384257

[pbi12774-bib-0008] Champion, A. , Hebrard, E. , Parra, B. , Bournaud, C. , Marmey, P. , Tranchant, C. and Nicole, M. (2009) Molecular diversity and gene expression of cotton ERF transcription factors reveal that group IXa members are responsive to jasmonate, ethylene and Xanthomonas. Mol. Plant Pathol. 10, 471–485. 19523101 10.1111/j.1364-3703.2009.00549.x PMC6640365

[pbi12774-bib-0009] Chen, Y.F. , Randlett, M.D. , Findell, J.L. and Schaller, G.E. (2002) Localization of the ethylene receptor ETR1 to the endoplasmic reticulum of Arabidopsis. J. Biol. Chem. 277, 19861–19866. 11916973 10.1074/jbc.M201286200

[pbi12774-bib-0010] Cheng, M.C. , Liao, P.M. , Kuo, W.W. and Lin, T.P. (2013) The Arabidopsis ETHYLENE RESPONSE FACTOR1 regulates abiotic stress‐responsive gene expression by binding to different cis‐acting elements in response to different stress signals. Plant Physiol. 162, 1566–1582. 23719892 10.1104/pp.113.221911 PMC3707555

[pbi12774-bib-0011] Duan, C. , Rio, M. , Leclercq, J. , Bonnot, F. , Oliver, G. and Montoro, P. (2010) Gene expression pattern in response to wounding, methyl jasmonate and ethylene in the bark of *Hevea brasiliensis* . Tree Physiol. 30, 1349–1359. 20660491 10.1093/treephys/tpq066

[pbi12774-bib-0012] Duan, C. , Argout, X. , Gébelin, V. , Summo, M. , Dufayard, J.‐F. , Leclercq, J. , Kuswanhadi *et al*. (2013) Identification of the *Hevea brasiliensis* AP2/ERF superfamily by RNA sequencing. BMC Genom. 14, 30. 10.1186/1471-2164-14-30 PMC3644242 23324139

[pbi12774-bib-0013] Ecker, J.R. and Solano, R. (2002) Ethylene‐response‐factor1 (erf1) in Plants. University of Pennsylvania, Philadelphia, Pennsylvania: Google Patents.

[pbi12774-bib-0014] Etchells, J.P. , Provost, C.M. and Turner, S.R. (2012) Plant vascular cell division is maintained by an interaction between PXY and ethylene signalling. PLoS Genet. 8, e1002997. 23166504 10.1371/journal.pgen.1002997 PMC3499249

[pbi12774-bib-0015] Fisher, D.B. (1968) Protein staining of ribboned epon sections for light microscopy. Histochemie, 16, 92–96. 4180491 10.1007/BF00306214

[pbi12774-bib-0016] Geigenberger, P. , Tiessen, A. and Meurer, J. (2011) Use of non‐aqueous fractionation and metabolomics to study chloroplast function in Arabidopsis. Methods Mol. Biol. 775, 135–160. 21863442 10.1007/978-1-61779-237-3_8

[pbi12774-bib-0017] Gomez‐Garay, A. , Lopez, J.A. , Camafeita, E. , Bueno, M.A. and Pintos, B. (2013) Proteomic perspective of Quercus suber somatic embryogenesis. J. Proteomics. 93, 314–325. 23770300 10.1016/j.jprot.2013.06.006

[pbi12774-bib-0018] Hao, B.‐Z. and Wu, J.‐L. (1982) Effects of wound (tapping) on laticifer differentiation in *Hevea brasiliensis* . Acta Bot. Sin. 24, 388–391.

[pbi12774-bib-0019] Hao, B.‐Z. and Wu, J.‐L. (1984) Acceleration of laticifer differentiation in *Hevea brasiliensis* by latex drainage. Chinese J. Trop. Crops, 5, 19–23.

[pbi12774-bib-0020] Hao, B.‐Z. and Wu, J.‐L. (2000) Laticifer differentiation in *Hevea brasiliensis*: induction by Exogenous Jasmonic Acid and Linolenic Acid. Ann. Bot. 85, 37–43.

[pbi12774-bib-0021] Hong, H. , Xiao, H. , Yuan, H. , Zhai, J. and Huang, X. (2015) Cloning and characterisation of JAZ gene family in *Hevea brasiliensis* . Plant Biol. 17, 618–624. 25399518 10.1111/plb.12288

[pbi12774-bib-0022] Jayashree, R. , Rekha, K. , Venkatachalam, P. , Uratsu, S.L. , Dandekar, A.M. , Kumari Jayasree, P. , Kala, R.G. *et al*. (2003) Genetic transformation and regeneration of rubber tree (*Hevea brasiliensis* Muell. Arg) transgenic plants with a constitutive version of an anti‐oxidative stress superoxide dismutase gene. Plant Cell Rep. 22, 201–209. 14551734 10.1007/s00299-003-0666-x

[pbi12774-bib-0023] Jo, L. , Dos Santos, A.L. , Bueno, C.A. , Barbosa, H.R. and Floh, E.I. (2014) Proteomic analysis and polyamines, ethylene and reactive oxygen species levels of Araucaria angustifolia (Brazilian pine) embryogenic cultures with different embryogenic potential. Tree Physiol. 34, 94–104. 24327423 10.1093/treephys/tpt102

[pbi12774-bib-0024] Kadir, S. , Von Weihe, M. and Al‐Khatib, K. (2007) Photochemical efficiency and recovery of photosystem II in grapes after exposure to sudden and gradual heat stress. J. Am. Soc. Hortic. Sci. 132, 764–769.

[pbi12774-bib-0025] Kepczynska, E. and Zielinska, S. (2011) Disturbance of ethylene biosynthesis and perception during somatic embryogenesis in *Medicago sativa* L. reduces embryos’ ability to regenerate. Acta Physiol. Plant. 33, 1969–1980.

[pbi12774-bib-0026] Lardet, L. , Piombo, G. , Oriol, F. , Dechamp, E. and Carron, M.P. (1999) Relations between biochemical characteristics and conversion ability in *Hevea brasiliensis* zygotic and somatic embryos. Can. J. Bot. 77, 1168–1177.

[pbi12774-bib-0027] Lardet, L. , Martin, F. , Dessailly, F. , Carron, M.P. and Montoro, P. (2007) Effect of exogenous calcium on post‐thaw growth recovery and subsequent plant regeneration of cryopreserved embryogenic calli of *Hevea brasiliensis* (Mull. Arg.). Plant Cell Rep. 26, 559–569. 17186244 10.1007/s00299-006-0278-3

[pbi12774-bib-0028] Lardet, L. , Dessailly, F. , Carron, M.P. , Rio, M.A. , Ferriere, N. and Montoro, P. (2009) Secondary somatic embryogenesis in *Hevea brasiliensis* (Mull. Arg.): an alternative process for long‐term somatic embryogenesis. J. Rubber Res. 12, 215–228.

[pbi12774-bib-0029] Leclercq, J. , Lardet, L. , Martin, F. , Chapuset, T. , Oliver, G. and Montoro, P. (2010) The green fluorescent protein as an efficient selection marker for *Agrobacterium tumefaciens*‐mediated transformation in *Hevea brasiliensis* (Mull. Arg). Plant Cell Rep. 29, 513–522. 20306052 10.1007/s00299-010-0840-x

[pbi12774-bib-0030] Leclercq, J. , Martin, F. , Sanier, C. , Clement‐Vidal, A. , Fabre, D. , Oliver, G. , Lardet, L. *et al*. (2012) Over‐expression of a cytosolic isoform of the HbCuZnSOD gene in *Hevea brasiliensis* changes its response to a water deficit. Plant Mol. Biol. 80, 255–272. 22814939 10.1007/s11103-012-9942-x

[pbi12774-bib-0031] Leclercq, J. , Szabolcs, T. , Martin, F. and Montoro, P. (2015) Development of a new pCAMBIA binary vector using Gateway(R) technology. Plasmid, 81, 50–54. 26210260 10.1016/j.plasmid.2015.07.003

[pbi12774-bib-0032] Licausi, F. , Pucciariello, C. and Perata, P. (2013) New role for an old rule: N‐end rule‐mediated degradation of ethylene responsive factor proteins governs low oxygen response in plants(F). J. Integr. Plant Biol. 55, 31–39. 23164408 10.1111/jipb.12011

[pbi12774-bib-0033] Lillie, R.D. and Ashburn, L.L. (1943) Supersaturated solutions of fat stains in dilute isopropanol for demonstration of acute fatty degeneration not shown by Herxheimer's technique. Arch. Pathol. 36, 432–440.

[pbi12774-bib-0034] Loh, S.C. , Thottathil, G.P. and Othman, A.S. (2016) Identification of differentially expressed genes and signalling pathways in bark of *Hevea brasiliensis* seedlings associated with secondary laticifer differentiation using gene expression microarray. Plant Physiol. Biochem. 107, 45–55. 27236227 10.1016/j.plaphy.2016.05.011

[pbi12774-bib-0035] Lorenzo, O. , Piqueras, R. , Sanchez‐Serrano, J.J. and Solano, R. (2003) ETHYLENE RESPONSE FACTOR1 integrates signals from ethylene and jasmonate pathways in plant defense. Plant Cell, 15, 165–178. 12509529 10.1105/tpc.007468 PMC143489

[pbi12774-bib-0036] Luquet, D. , Clément‐Vidal, A. , Fabre, D. , This, D. and Sonderegger, N. (2008) Orchestration of transpiration, growth and carbohydrate dynamics in rice during a dry‐down cycle. Funct. Plant Biol. 35, 689–704. 32688823 10.1071/FP08027

[pbi12774-bib-0037] Manners, J.M. , Penninckx, I.A. , Vermaere, K. , Kazan, K. , Brown, R.L. , Morgan, A. , Maclean, D.J. *et al*. (1998) The promoter of the plant defensin gene PDF1.2 from Arabidopsis is systemically activated by fungal pathogens and responds to methyl jasmonate but not to salicylic acid. Plant Mol. Biol. 38, 1071–1080. 9869413 10.1023/a:1006070413843

[pbi12774-bib-0038] Mao, J.L. , Miao, Z.Q. , Wang, Z. , Yu, L.H. , Cai, X.T. and Xiang, C.B. (2016) Arabidopsis ERF1 mediates cross‐talk between ethylene and auxin biosynthesis during primary root elongation by regulating ASA1 expression. PLoS Genet. 12, e1006076. 26745809 10.1371/journal.pgen.1005760 PMC4706318

[pbi12774-bib-0039] Masato, O. (1980) An improved method for determination of l‐ascorbic acid and l‐dehydroascorbic acid in blood plasma. Clin. Chim. Acta, 103, 259–268. 7398071 10.1016/0009-8981(80)90144-8

[pbi12774-bib-0040] Mialet‐Serra, I. , Clement, A. , Sonderegger, N. , Roupsard, O. , Jourdan, C. , Labouisse, J.P. and Dingkuhn, M. (2005) Assimilate storage in vegetative organs of coconuts (Cocos nucifera). Expl. Agric. 41, 161–174.

[pbi12774-bib-0041] Montoro, P. , Lagier, S. , Baptiste, C. , Marteaux, B. , Pujade‐Renaud, V. , Leclercq, J. and Alemanno, L. (2008) Expression of the HEV2.1 gene promoter in transgenic Hevea brasiliensis. Plant Cell, Tissue Organ Cult. 94, 55–63.

[pbi12774-bib-0042] Nakano, T. , Suzuki, K. , Fujimura, T. and Shinshi, H. (2006) Genome‐wide analysis of the ERF gene family in Arabidopsis and rice. Plant Physiol. 140, 411–432. 16407444 10.1104/pp.105.073783 PMC1361313

[pbi12774-bib-0043] Onate‐Sanchez, L. and Singh, K.B. (2002) Identification of Arabidopsis ethylene‐responsive element binding factors with distinct induction kinetics after pathogen infection. Plant Physiol. 128, 1313–1322. 11950980 10.1104/pp.010862 PMC154259

[pbi12774-bib-0044] Pierik, R. , Tholen, D. , Poorter, H. , Visser, E. and Voesenek, L. (2006) The Janus face of ethylene: growth inhibition and stimulation. Trends Plant Sci. 11, 176–183. 16531097 10.1016/j.tplants.2006.02.006

[pbi12774-bib-0045] Pirrello, J. , Leclercq, J. , Dessailly, F. , Rio, M. , Piyatrakul, P. , Kuswanhadi, K. , Tang, C. *et al*. (2014) Transcriptional and post‐transcriptional regulation of the jasmonate signalling pathway in response to abiotic and harvesting stress in *Hevea brasiliensis* . BMC Plant Biol. 14, 17. 25443311 10.1186/s12870-014-0341-0 PMC4274682

[pbi12774-bib-0046] Piyatrakul, P. , Putranto, R.A. , Martin, F. , Rio, M. , Dessailly, F. , Leclercq, J. , Dufayard, J.F. *et al*. (2012) Some ethylene biosynthesis and AP2/ERF genes reveal a specific pattern of expression during somatic embryogenesis in *Hevea brasiliensis* . BMC Plant Biol. 12, 244. 23268714 10.1186/1471-2229-12-244 PMC3561283

[pbi12774-bib-0047] Piyatrakul, P. , Yang, M. , Putranto, R.A. , Pirrello, J. , Dessailly, F. , Hu, S. , Summo, M. *et al*. (2014) Sequence and expression analyses of ethylene response factors highly expressed in latex cells from *Hevea brasiliensis* . PLoS One, 9, e99367. 24971876 10.1371/journal.pone.0099367 PMC4074046

[pbi12774-bib-0048] Pré, M. , Atallah, M. , Champion, A. , De Vos, M. , Pieterse, C.M. and Memelink, J. (2008) The AP2/ERF domain transcription factor ORA59 integrates jasmonic acid and ethylene signals in plant defense. Plant Physiol. 147, 1347–1357. 18467450 10.1104/pp.108.117523 PMC2442530

[pbi12774-bib-0049] Putranto, R.A. , Duan, C. , Kuswanhadi , Chaidamsari, T. , Rio, M. , Piyatrakul, P. , Herlinawati, E. *et al*. (2015a) Ethylene response factors are controlled by multiple harvesting stresses in *Hevea brasiliensis* . PLoS One, 10, e0123618. 25906196 10.1371/journal.pone.0123618 PMC4408094

[pbi12774-bib-0050] Putranto, R.A. , Herlinawati, E. , Rio, M. , Leclercq, J. , Piyatrakul, P. , Gohet, E. , Sanier, C. *et al*. (2015b) Involvement of ethylene in the latex metabolism and tapping panel dryness of *Hevea brasiliensis* . Int. J. Mol. Sci. 16, 17885–17908. 26247941 10.3390/ijms160817885 PMC4581227

[pbi12774-bib-0051] Putranto, R.A. , Herlinawati, E. , Rio, M. , Leclercq, J. , Piyatrakul, P. , Gohet, E. , Sanier, C. *et al*. (2015c) Involvement of ethylene in the latex metabolism and tapping panel dryness of *Hevea brasiliensis* . Int. J. Mol. Sci. 16, 17885–17908. 26247941 10.3390/ijms160817885 PMC4581227

[pbi12774-bib-0052] Queval, G. and Noctor, G. (2007) A plate reader method for the measurement of NAD, NADP, glutathione, and ascorbate in tissue extracts: application to redox profiling during Arabidopsis rosette development. Anal. Biochem. 363, 58–69. 17288982 10.1016/j.ab.2007.01.005

[pbi12774-bib-0053] Ray, J.D. , Gesch, R.W. , Sinclair, T.R. and Hartwell Allen, L. (2002) The effect of vapor pressure deficit on maize transpiration response to a drying soil. Plant Soil, 239, 113–121.

[pbi12774-bib-0054] Sanier, C. , Oliver, G. , Clement‐Vidal, A. , Fabre, D. , Lardet, L. and Montoro, P. (2013a) Influence of water deficit on the physiological and biochemical parameters of in vitro plants from *Hevea brasiliensis* Clone PB 260. J. Rubber Res. 16, 61–74.

[pbi12774-bib-0055] Sanier, C. , Oliver, G. , Clément Demange, A. , Fabre, D. , Lardet, L. and Montoro, P. (2013b) Influence of water deficit on physiological parameters and antioxidant system on in vitro plants from the *Hevea brasiliensis* clone PB260. J. Rubber Res. 16, 61–74.

[pbi12774-bib-0056] Shi, Z.Q. and Hu, Z.H. (1965) Method for making slide of rubber containing plant tissue. Acta Bot. Sin. 13, 179–183.

[pbi12774-bib-0057] Silpi, U. , Lacointe, A. , Kasempsap, P. , Thanysawanyangkura, S. , Chantuma, P. , Gohet, E. , Musigamart, N. *et al*. (2007) Carbohydrate reserves as a competing sink: evidence from tapping rubber trees. Tree Physiol. 27, 881–889. 17331906 10.1093/treephys/27.6.881

[pbi12774-bib-0058] Sinclair, T. and Ludlow, M. (1986) Influence of soil water supply on the plant water balance of four tropical grain legumes. Funct. Plant Biol. 13, 329–341.

[pbi12774-bib-0059] Solano, R. , Stepanova, A. , Chao, Q. and Ecker, J.R. (1998) Nuclear events in ethylene signaling: a transcriptional cascade mediated by ETHYLENE‐INSENSITIVE3 and ETHYLENE‐RESPONSE‐FACTOR1. Genes Dev. 12, 3703–3714. 9851977 10.1101/gad.12.23.3703 PMC317251

[pbi12774-bib-0060] Strasserf, R.J. , Srivastava, A. and Govindjee, G. (1995) Polyphasic chlorophyll a fluorescence transient in plants and Cyanobacteria. Photochem. Photobiol. 61, 32–42.

[pbi12774-bib-0061] Strauss, A.J. , Krüger, G.H.J. , Strasser, R.J. and Heerden, P.D.R.V. (2006) Ranking of dark chilling tolerance in soybean genotypes probed by the chlorophyll a fluorescence transient O‐J‐I‐P. Environ. Exp. Bot. 56, 147–157.

[pbi12774-bib-0062] Szabados, L. and Savoure, A. (2010) Proline: a multifunctional amino acid. Trends Plant Sci. 15, 89–97. 20036181 10.1016/j.tplants.2009.11.009

[pbi12774-bib-0063] Tian, W. , Shi, M. , Yu, F. , Wu, J. , Hao, B. and Cui, K. (2003) Localized effects of mechanical wounding and exogenous jasmonic acid on the induction of secondary laticifer differentiation in relation to the distribution of jasmonic acid in *Hevea brasiliensis* . Acta Bot. Sin. 45, 1366–1372.

[pbi12774-bib-0064] Tian, W.M. , Yang, S.G. , Shi, M.J. , Zhang, S.X. and Wu, J.L. (2015) Mechanical wounding‐induced laticifer differentiation in rubber tree: an indicative role of dehydration, hydrogen peroxide, and jasmonates. J. Plant Physiol. 182, 95–103. 26070085 10.1016/j.jplph.2015.04.010

[pbi12774-bib-0065] Udvardi, M.K. , Czechowski, T. and Scheible, W.R. (2008) Eleven golden rules of quantitative RT‐PCR. Plant Cell, 20, 1736–1737. 18664613 10.1105/tpc.108.061143 PMC2518243

[pbi12774-bib-0066] Vahala, J. , Felten, J. , Love, J. , Gorzsás, A. , Gerber, L. , Lamminmäki, A. , Kangasjärvi, J. *et al*. (2013) A genome‐wide screen for ethylene‐induced ethylene response factors (ERFs) in hybrid aspen stem identifies ERF genes that modify stem growth and wood properties. New Phytol. 200, 511–522. 23815789 10.1111/nph.12386

[pbi12774-bib-0067] Wu, J.‐L. , Hao, B.‐Z. and Tan, H.‐Y. (2002) Wound‐induced laticifer differentiation in *Hevea brasiliensis* shoots mediated by jasmonic acid. J. Rubber Res. 5, 53–63.

[pbi12774-bib-0068] Wu, H.L. , Yu, B. , Cheng, Q.Q. , Zeng, R.Z. , Duan, C.F. , Nie, Z.Y. and Li, Y. (2010) Cloning and Characterization of Jasmonic Acid‐Induced AP2/EREBP Genes in Laticifer from Rubber Tree (*Hevea brasiliensis* Muell. Arg.). Chin. Agric. Sci. Bull. 26, 287–293.

[pbi12774-bib-0069] Zhang, S.X. and Tian, W.M. (2015) Cross talk between cytokinin and jasmonates in regulating the secondary laticifer differentiation in rubber tree (*Hevea brasiliensis* Muell. Arg.). J. Rubber Res. 18, 38–48.

[pbi12774-bib-0070] Zhang, S.X. , Wu, S.H. , Chen, Y.Y. and Tian, W.M. (2015) Analysis of Differentially Expressed Genes Associated with coronatine‐induced laticifer differentiation in the rubber tree by subtractive hybridization suppression. PLoS One, 10, e0132070. 26147807 10.1371/journal.pone.0132070 PMC4493031

