# Supplementary material for: Overexpression of Hevea brasiliensis ethylene response factor HbERF‐IXc5 enhances growth and tolerance to abiotic stress and affects laticifer differentiation
Source: Plant Biotechnol J. 2017 Sep 2;16(1):322–36. doi: 10.1111/pbi.12774 (PMC5785357; doi:10.1111/pbi.12774)
Supplement: Supplementary file 1 — Figure S1 Comparison of latex cell‐specific staining using (a) iodine bromide treatment (Shi and Hu, 1965) and (b) oil red O (Montoro et al., 2008). Latex cells (LC) are specified by arrows and appear in brown and red for the two stainings, respectively. [file PBI-16-322-s002.pptx]

## Slide 1
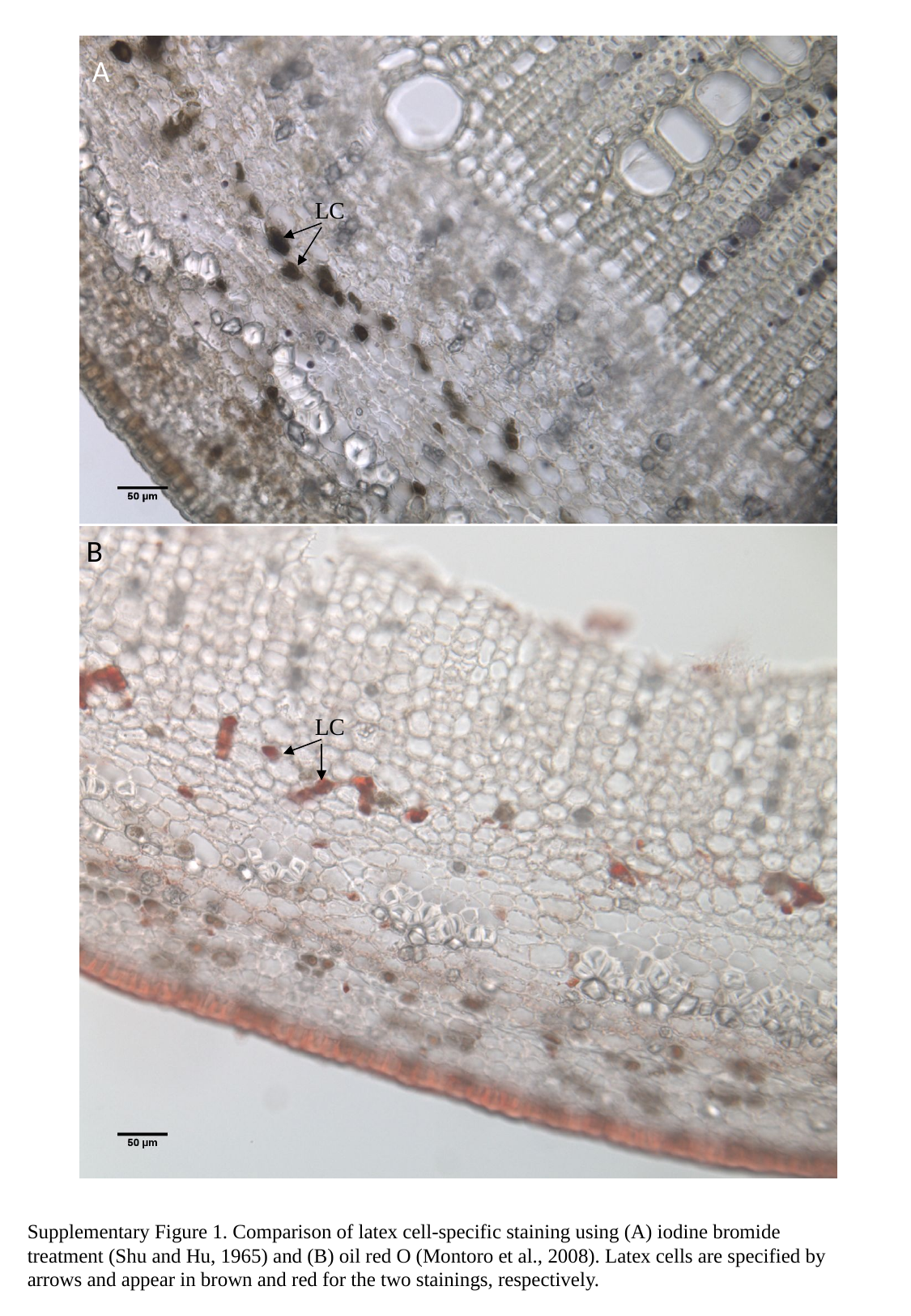

A
LC
B
LC
Supplementary Figure 1. Comparison of latex cell-specific staining using (A) iodine bromide treatment (Shu and Hu, 1965) and (B) oil red O (Montoro et al., 2008). Latex cells are specified by arrows and appear in brown and red for the two stainings, respectively.
